# An early female lethal system of the New World screwworm, *Cochliomyia hominivorax*, for biotechnology-enhanced SIT

**DOI:** 10.1186/s12863-020-00948-x

**Published:** 2020-12-18

**Authors:** Carolina Concha, Ying Yan, Alex Arp, Evelin Quilarque, Agustin Sagel, Adalberto Pérez de León, W. Owen McMillan, Steven Skoda, Maxwell J. Scott

**Affiliations:** 1 Panama-United States Commission for the Eradication and Prevention of Screwworm (COPEG), Pacora, Panama; 2 Smithsonian Tropical Research Institute, Apartado 0843-03092, Panama City, Panama; 3 Department for Insect Biotechnology in Plant Protection, Justus-Liebig-University Gießen, Winchesterstraße 2, 35394 Gießen, Germany; 4 USDA-ARS, Screwworm Research Site, Apartado 0816-07636, Pacora, Panama; 5 Knipling-Bushland U.S. Livestock Insects Research Laboratory, 2700 Fredericksburg Rd, Kerrville, TX 78028 USA; 6 Department of Entomology and Plant Pathology, North Carolina State University, Campus Box 7613, Raleigh, NC 27695-7613 USA

**Keywords:** Transgenic sexing strain, Pest control, Female lethal strain

## Abstract

**Background:**

The New World Screwworm fly (NWS), *Cochliomyia hominivorax*, is an ectoparasite of warm-blooded animals and a major pest of livestock in parts of South America and the Caribbean where it remains endemic. In North and Central America it was eradicated using the Sterile Insect Technique (SIT). A control program is managed cooperatively between the governments of the United States and Panama to prevent the northward spread of NWS from infested countries in South America. This is accomplished by maintaining a permanent barrier through the release of millions of sterile male and female flies in the border between Panama and Colombia. Our research team demonstrated the utility of biotechnology-enhanced approaches for SIT by developing a male-only strain of the NWS. The strain carried a single component tetracycline repressible female lethal system where females died at late larval/pupal stages. The control program can be further improved by removing females during embryonic development as larval diet costs are significant.

**Results:**

The strains developed carry a two-component system consisting of the *Lucilia sericata bottleneck* gene promoter driving expression of the tTA gene and a tTA-regulated *Lshid* proapoptotic effector gene. Insertion of the sex-specifically spliced intron from the *C. hominivorax transformer* gene within the *Lshid* gene ensures that only females die when insects are reared in the absence of tetracycline. In several double homozygous two-component strains and in one “All-in-one” strain that had both components in a single construct, female lethality occurred at the embryonic and/or first instar larval stages when raised on diet without tetracycline. Laboratory evaluation for phenotypes that are relevant for mass rearing in a production facility revealed that most strains had fitness characteristics similar to the wild type J06 strain that is currently reared for release in the permanent barrier. Testing of an “All in one” strain under mass rearing conditions showed that the strain maintained the fitness characteristics observed in small-scale rearing.

**Conclusions:**

The early female lethal strains described here could be selected by the NWS Control Program for testing at large scale in the production facility to enhance the efficiency of the NWS eradication program.

**Supplementary Information:**

**Supplementary information** accompaies this paper at 10.1186/s12863-020-00948-x.

## Background

Management of insect pests with a strong economic impact on crop and livestock production has been an ongoing global concern [1]. There is a growing need for alternatives to the use of pesticides, some of which have been banned from markets due to the presence of remaining residues on products like wool or fruits. Pesticide residues can also impact the environment by affecting non-target species [2, 3]. In response to this demand, the Sterile Insect Technique (SIT) has become widely used as an environmentally friendly and species-specific method of pest control [4, 5]. This technique involves the mass rearing and field dispersal of millions of male insects sterilized by irradiation, which compete with wild-type males to mate with fertile females in the field. As a consequence, females produce no viable offspring, thus gradually reducing insect populations over time. This non-chemical pest control method was originally developed to reduce populations of the New World screwworm (NWS), *Cochliomyia hominivorax* (Diptera, Calliphoridae), in the United States, where it succeeded in eradicating this ectoparasite of warm-blooded animals from the whole country and later from Mexico and Central America [4, 6, 7]. After this landmark success, SIT was adapted to manage effectively the populations of other economically important agricultural insect pests such as the Medfly and the Mexican fruit fly [8].

Adult female NWS flies lay their eggs on wounds or natural openings in the skin, like the area around the eyes or mouth of animal hosts [9]. Upon hatching from the eggs, the larvae, or screwworms, develop by feeding on host soft tissues. Heavy infestation can result in death of the host. Originally endemic in tropical and subtropical regions of the American continent, this insect caused damages of millions of dollars every year to the livestock industries of North and Central America until an area-wide pest management program with an SIT component, led by the United States Department of Agriculture (USDA), achieved its eradication from the United States in the 1960s [6, 10]. Extension of the program resulted in eradication of the NWS from the whole American continent north of Colombia by 2001. Currently, the Commission for the Eradication and Prevention of Screwworms (COPEG), managed cooperatively between USDA and the Panama Ministry of Agriculture and Livestock (MIDA), mass rears, sterilizes by irradiation, and releases millions of sterile NWS across the border between Panama and Colombia to prevent its re-introduction from South American countries [7], where it is still a major economic pest [11]. The NWS outbreak that occurred in Florida in 2016 highlighted why this insect is considered a high-consequence foreign animal pest in countries where it was eradicated [12].

A continued effort of USDA-COPEG includes performing research to increase efficiencies in NWS eradication and prevention. Current operations involve sterilization and field release of males and females, although only male NWS are effective in suppressing local populations. Sterile females co-released with males tend to distract them from mating with fertile females in the field, which increases the number of insects required for efficient population suppression. It has been shown in other SIT programs, such as the Medfly control program in Guatemala, that male-only releases may be 3–5 times more effective at reducing local populations than bi-sexual sterile releases [13]. Thus, less sterile insects are required to suppress the target population, an economic benefit that makes it desirable to develop such strains for the NWS eradication and prevention program.

We previously developed a conditional female lethal transgenic strain of NWS based on the overexpression of the tetracycline repressible transactivator (tTA) in females [14]. This autoregulatory system consists of the tTA gene, a core promoter and upstream enhancer consisting of multiple copies of the tTA binding site (tetO or TRE). tTA is a potent activator of transcription and consists of the DNA binding domain of the *E. coli tet* repressor fused to the transcription activation domain of the HSV1 virus VP16 protein [15]. Overexpression of tTA protein causes lethality, which is possibly due to “transcriptional squelching” or interference with ubiquitin-dependent proteolysis [16]. Binding of tTA to the tet operator (tetO) is strongly inhibited by addition of tetracycline to the diet, providing a simple switch-off system [15]. The system is made female specific by introducing the sex-specifically spliced first intron of the NWS *transformer* gene (*Chtra*) [17] after the translation start codon of tTA, such that only females can make a functional tTA protein. Ten homozygous female lethal strains were produced in the laboratory expressing this system and some strains exhibited mass rearing and fitness characteristics that made them suitable for a control program. Although a sexing strain of this kind may enhance program efficiencies by introducing important savings in production and dispersal costs [14], this genetic system removes the females at the third instar larvae/pupa stage, after they have consumed larval diet. Additional savings in production costs might be obtained with a sexing strain that removes females from mass rearing before they start feeding.

**Fig. 1:**
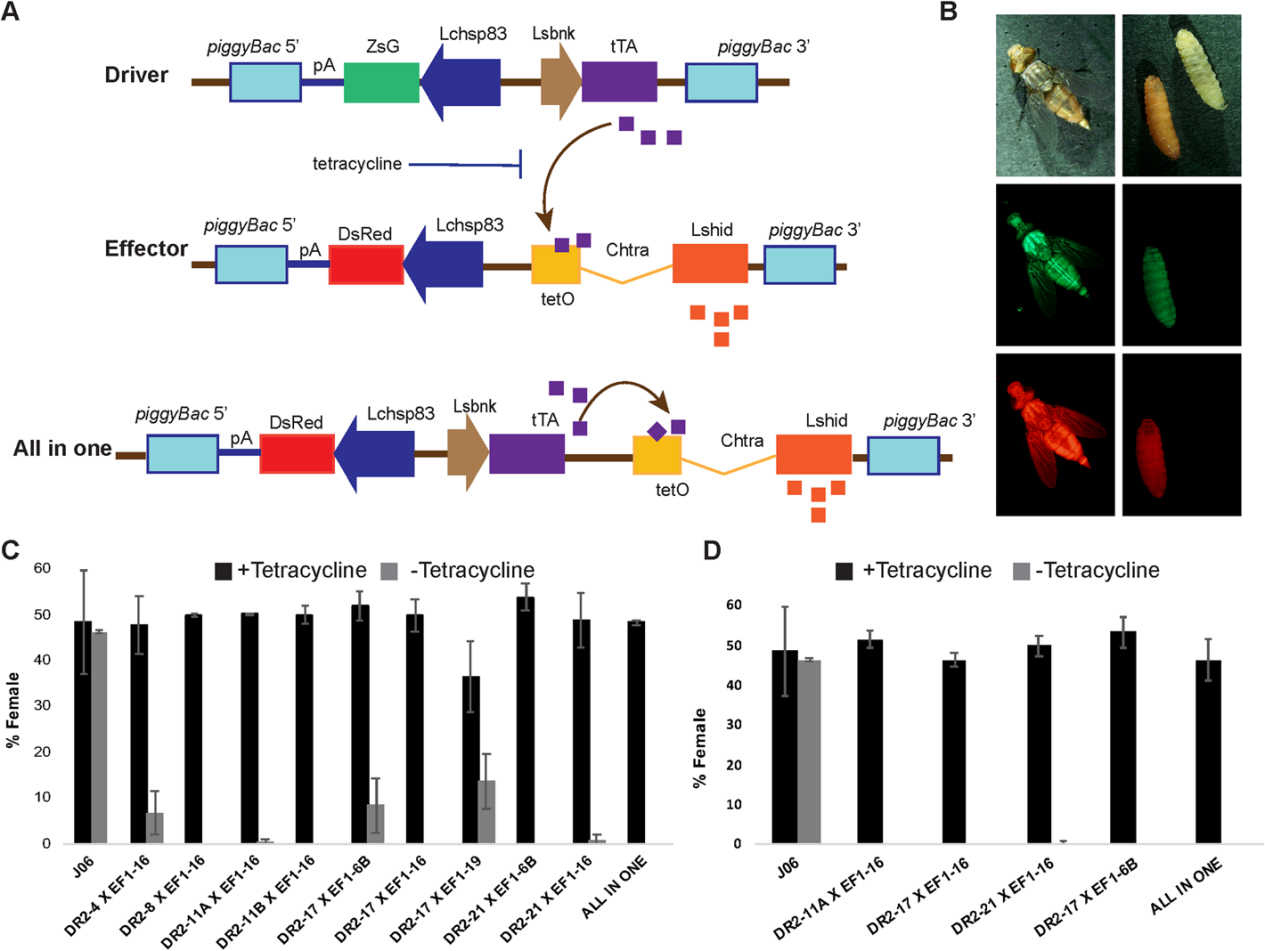
A transgenic female early lethal system for control of the New World screwworm. **a** Schematic diagram of the genetic constructs used to create female early lethal strains in NWS. A two-component system consisting of a Driver construct that expresses tTAo protein under the control of the *Lucilia sericata bottleneck* (*Lsbnk*) promoter and an Effector construct, composed of 21 copies of the tTA binding site, tetO, upstream of the *L. sericata* apoptoti*c hid* gene. The *hid* gene contained two mutations (*Lshid*^*Ala2*^) that should prevent inhibition by MAPK and thus increase activity [22]. The All-in-one construct contains both Driver and Effector cassettes in the same transformation vector. **b** Two component transgenic lines express both red and green fluorescent markers at the late larval, pupal and adult stages. **c** Heterozygous and **d** homozygous female lethality when insects were reared in the absence of tetracycline in the diet. Eight pairs of flies were used for each test, and error bars show the standard error of the mean (*n* = 3)

Transgenic embryonic female lethal systems have been developed for tephritid fruit flies [18, 19] and blow flies [20, 21], based on a two-component genetic system. This system consists on a Driver construct composed of an early active embryonic promoter controlling the expression of the tTA gene and an Effector construct with a tTA-regulated pro-apoptotic gene. The system is made female specific in a similar way to the single component system, by the addition of the first *C. hominivorax tra* intron after the start codon of the proapoptotic gene (Fig. 1a). Transgenic strains are created carrying either of the two constructs and, then homozygous lines for Driver and Effector are crossed to each other. The genetic system is complete when the strains are made homozygous for both components. As an antidote for this system, tetracycline can bind tTA and prevent its binding to tetO, which will prevent the synthesis of the apoptotic protein. Hence, homozygous strains reared in diet lacking tetracycline will only develop males while those reared in diet containing tetracycline will develop both sexes. In this study, we report that NWS early female lethal strains were created based on this system. These strains are suitable for testing at large scale mass rearing conditions and could be developed within the mass production facility to enhance the efficiency of the NWS eradication program.

## Results

### An early lethal transgenic sexing system for *C. hominivorax*

A series of Driver and Effector constructs were previously designed for their application to a range of blow flies, including the Australian sheep blow fly *Lucilia cuprina*, the European green blow fly *L. sericata*, the Old World primary screwworm *Chrysomya bezziana*, the secondary screwworm *C. macellaria*, and the NWS [20, 23]. The DR2 Driver had the desired characteristics of high expression in early embryos and low expression at later stages including adult female ovaries [20, 23]. The Driver contained the *L. sericata bottleneck* (*Lsbnk*) promoter upstream of a codon optimized tTAo gene in a *piggyBac* transformation vector with a ZsGreen marker gene. Following *C. hominivorax* embryo microinjection with the DR2 construct and a *piggyBac* helper plasmid, nine transgenic lines were obtained and bred to homozygosity by screening for strong green fluorescence (Fig. 1a, b). The Effector *piggyBac* vector (EF1) contained a phosphomutated version of the proapoptotic *L. sericata hid* gene (*Lshid*^*Ala2*^) under the control of tetO-hsp70 enhancer-promoter. In the protein encoded by the *Lshid*^*Ala2*^ gene, two amino acids were changed to Alanine which should prevent phosphorylation and inhibition by MAP kinase [20, 22]. Five EF1 transgenic lines were obtained and bred to homozygosity by screening for strong red fluorescence as the *piggyBac* vector contained the *Lchsp83-DsRedEx2* marker (Fig. 1a, b). A preliminary crossing experiment showed that EF1#6B and EF1#16 were the only transgenic lines with a strong female killing effect (data not shown). To test the performance of DR2 lines, heterozygous lethality tests were carried out by crossing the nine homozygous DR2 lines with the EF1#16 line. All nine combinations of DR2/ EF1#16 showed high female lethality (range of 0 to 14% females) on standard diet which lacks tetracycline (additional file 1, and data not shown). Four of the DR2 Driver lines (#8, #11B, #17 and #21) were dominant female lethal in combination with an effector (Fig. 1c and Additional file 1). In other words, in four of the combinations tested a single copy of each component (double heterozygous) was enough to produce 100% female lethality when insects were reared in diet without tetracycline. Next, we bred all nine combinations of DR2/EF1 to double homozygosity (DH) (Fig. 1c), by selecting for the bright fluorescent red and green third instar larvae at each generation. Preliminary observations (e.g. fecundity) identified four promising strains that were then maintained in the laboratory for over 20 generations. These lines were 100% female lethal when reared in diet without tetracycline (Fig. 1d and Additional file 1).

We also made transgenic lines with an “All-in-one” (TD1) construct that had both the *Lsbnk-tTA* Driver and tetO-*Lshid*^*Ala2*^ Effector cassettes in a single *piggyBac* vector [20]. As each transgene could carry a fitness cost due to the possibility of insertional mutations, a strain with a single transgene could potentially have lower fitness costs than a double homozygous strain. Further, it is easier to breed to homozygosity with a single marker gene. Consequently, three transgenic lines were obtained and were bred to homozygosity by screening for bright red fluorescence (Fig. 1a). However, two of the TD1 homozygous lines were lost during rearing due to low fitness but the third line (#12) was homozygous viable, fertile and showed dominant female lethality when reared in diet lacking tetracycline (Fig. 1c, d).

### Transgenic females are removed from rearing as embryos or first instar larvae

The DH strains were initially reared on diet containing 100 μg/mL tetracycline as used previously with *L. cuprina* DH strains [20]. However, we noticed that there was great variation between the transgenic lines in some fitness characteristics such as the percentage of eggs that developed into pupae, the adult emergence ratio and male/female ratios after emergence (data not shown). We considered this was possibly due to insufficient suppression of the female lethal system. Thus, we next performed tetracycline dosage studies to determine the optimal dose for rearing higher numbers of insects with a balanced male/female ratio (Additional file 2). We found the optimal conditions for rearing the DH strains was with larval diet containing 200 μg/mL tetracycline as this gave the highest numbers of eggs laid and development to pupae (biological yield), and percentage of emergence. For the single component sexing system that we previously developed in NWS [14], adult females were viable and produced male-only offspring when their eggs were reared on larval food without tetracycline. In contrast, under the same rearing conditions, the females of some DH strains were sterile and short-lived. A similar phenomenon was previously observed in *L. cuprina* two-component systems that used the *Lsbnk-tTA* driver [20], which were shown to express tTA in ovaries [21, 23], thus activating the effector gene. Adding tetracycline in the drinking water of adult insects (10 μg/mL) for one or two days after eclosion at least partially rescued the viability and fertility of females for the four DH strains (additional file 3). It would appear that in the NWS DH strains, tTA might have also been expressed in the ovaries but that the additional supply of tetracycline efficiently prevented *Lshid*^*Ala2*^ expression.

**Fig. 2:**
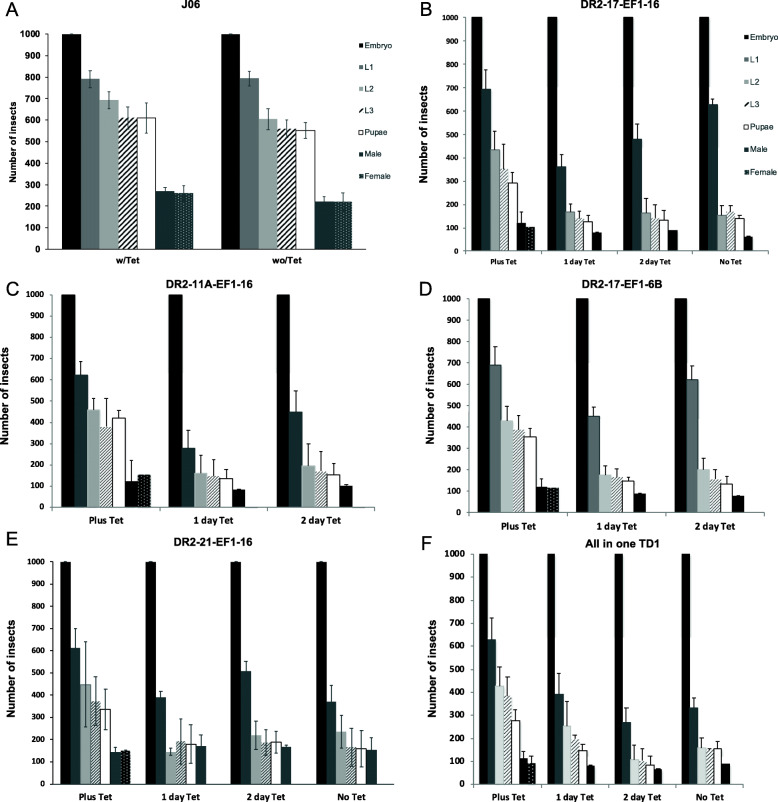
Transgenic NWS lines show early female lethality. Female specific lethality of four double homozygous two-component lines and an All-in-one line (TD1) was studied at each developmental stage from egg to adult. 1000 eggs were placed in larval diet, containing the following tetracycline (Tet) regimens: 200 μg/mL Tet in the larval diet and 10 μg/mL in adult drinking water, no Tet in the larval diet and 10 μg/mL in the drinking water for one or two days, and no Tet in the larval or adult diet. The number of first, second and third instar larvae, pupae and adult male and females were counted. **a** The wild type J06 strain on diet lacking tetracycline, **b** DR2–17/EF1–16, **c** DR2-11A/EF1–16, **d** DR2–17/EF1-6B, **e** DR2–21/EF1–16 and **f** All-in-one or TD1. All experiments were repeated at least three times and the average and standard deviation (error bars) are shown

Although limited feeding of tetracycline to adult females of the four DH strains improved their viability and fertility, it was possible that the females could pass tetracycline on to their eggs. If so, this would turn off the early female-lethality system in developing embryos. Therefore, we performed staged lethality tests under different tetracycline feeding regimens to determine the stage of development at which females died (Fig. 2). As a control, we also tested the parental wild type strain J06, in diet containing or lacking tetracycline. Each individual experiment was repeated at least three times. We found that under permissive tetracycline conditions (200 μg/mL tetracycline was provided in larval diet and parental water), strains DR2#17/EF1#6B (*P* = 0.010, One-way ANOVA), DR2#17 /EF1#16 (*P* = 0.016, One-way ANOVA), and TD1#12 (*P* = 0.025, One-way ANOVA) produced significantly fewer adults per one thousand eggs than the wild type parental strain J06. However, there was no significant difference in adult production between DR2#11A/EF1#16 (*P* = 0.060, One-way ANOVA) or DR2#21/ EF1#16 (*P* = 0.212, One-way ANOVA) and J06 (Fig. 2). On a restrictive tetracycline diet (larval diet without tetracycline and parental water either without tetracycline or at 10 μg/mL for one or two days), no adult females were obtained from any of the transgenic female lethal strains, suggesting that any maternal tetracycline passed to the offspring was not enough to suppress the lethal system. Importantly, most of the transgenic strains showed a reduction of about half the number of second instar larvae with respect to the number of first instar larvae hatched from one thousand eggs, suggesting that females were dying as first instar larvae. The exception to this was the DR2-11A/EF1–16 transgenic strain (Fig. 2c), in which the number of first instar larvae hatched in larval diet lacking tetracycline and with one day of tetracycline in the parental water, was less than half the number of eggs that were seeded. This could indicate that females were dying at the embryo stage. It is also possible that there was some male lethality in this strain. Interestingly, for two of the strains, DR2–21/EF1–16 and TD1, it did not appear necessary to add tetracycline to the parental water as females were fertile without additional tetracycline and produced approximately the same number of adult males per 1000 eggs as the other transgenic strains (t = − 1503, *P* = 0.207, d.f. = 4, t-test). Further, the numbers of males were approximately equal to those produced under permissive tetracycline conditions for both DR2–21/EF1–16 (t = − 0,237, *P* = 0.824, d.f. = 4, t-test) and TD1(t = 0.566, *P* = 0.602, d.f. = 4, t-test) (Fig. 2b, e, f). Hence, these two strains could be considered for mass rearing in an insect production plant, as the water treatment given to them would be the same throughout the rearing process, simplifying their management.

### Fitness of early female lethal strains for mass rearing

**Fig. 3:**
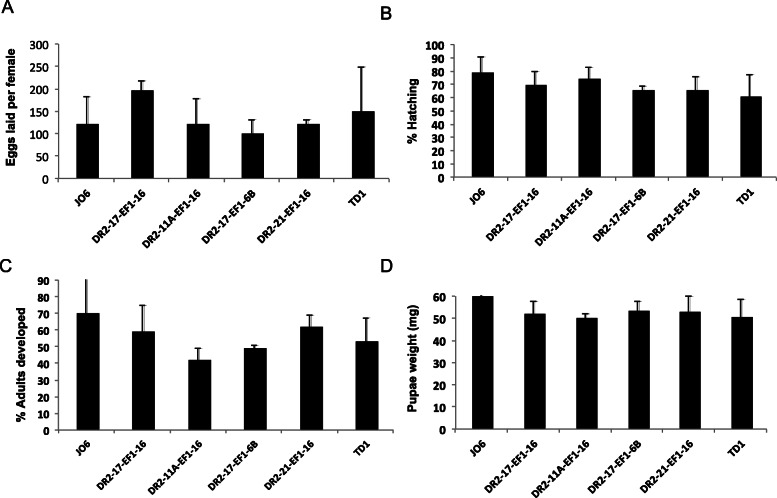
Fitness tests of transgenic female early lethal strains. Four homozygous two-component strains and an All-in-one strain (TD1) were evaluated for fitness parameters relevant to mass rearing and compared to the wild type J06 strain that is currently reared at the COPEG facility. These fitness parameters are **a** Average number of eggs laid per cage, **b** The percentage of eggs that hatch into first instar larvae, **c** The percentage eggs that develop into adults, and **d** the average weight of the pupae. These tests were performed with 200 μg/mL tetracycline in larval diet and 10 μg/mL in the adult drinking water. All experiments were repeated at least three times, with the average and standard deviation (error bars) shown

One of the major aims of developing an early female lethal strain of the NWS was to reduce the costs of production in a mass rearing facility and to improve the efficiency of the control program by rearing fewer insects for the general operations of the production plant. For this reason, we evaluated the fitness of our strains for characteristics relevant to mass rearing. In the majority of the transgenic strains we observed similar fitness characteristics to the parental J06 strain when insects were reared with diet containing tetracycline (Fig. 3 a-d). For all the characteristics studied, the DH strains DR2–17/EF1–16, DR2–21/EF1–16 and TD1 had the highest values, similar to the control strain J06. Specifically, there was no significant difference in the number of eggs (*P* = 0.052, One-Way ANOVA; Fig. 3a), average pupae weight (*P* = 0.495, One-Way ANOVA; Fig. 3d) and pupae development rate (*P* = 0.100, One-Way ANOVA; Fig. 3c) produced from each cage from all tested strains. All strains had similar hatch rate (*P* > 0.05, One-Way ANOVA), except DR2–17/ EF1-6B (*P* = 0.039, One-Way ANOVA) which showed significantly lower hatch rate than that of J06 (Fig. 3b).

**Fig. 4:**
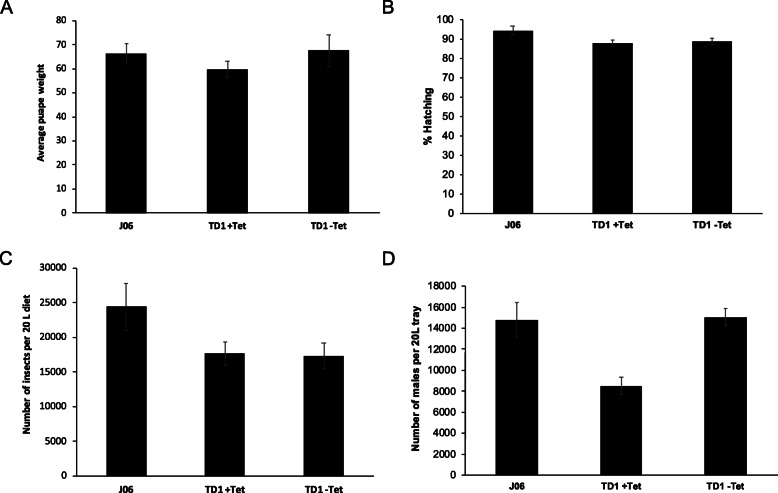
Fitness tests of an All-in-one strain under mass rearing conditions. Twice the number of eggs of the TD1 strain were seeded on diet without tetracycline than were seeded in diet containing tetracycline or relative to J06 on diet without tetracycline. **a** The average weight of one pupa. **b** The percentage of first instar larvae hatched from eggs. **c** The number of adults that develop from 20 L of larval diet are shown. **d** The number of males that develop from 20 L of larval diet are shown under the same conditions as in **c**

These fitness tests were performed at a laboratory scale, where 100 mg of eggs were seeded on larval diet. In order to have a better understanding of how any of these early female lethal strains would behave on a mass rearing scale, we selected the TD1 strain for rearing in 20 L trays of larval diet, which is the unit of rearing at the mass production facility. In these large-scale conditions, we observed no significant difference in the average pupae weight (*P* = 0.2, One-Way ANOVA; Fig. 4a) or the adult emerge rate (*P* = 0.347, One-Way ANOVA; additional file 4a) of the TD1 strain (with or without tetracycline) and J06 strain (without tetracycline). There was no significant difference in the hatching rate from TD1 strain with and without tetracycline (*P* = 0.552, One-Way ANOVA;), but they were both significantly lower than that of J06 strain (*P* < 0.05, One-Way ANOVA; Fig. 4b). TD1 reared on 20 L of diet with tetracycline, produced significantly fewer adults than the J06 strain from the same starting number of eggs (Fig. 4c; *P* = 0.009, One-Way ANOVA), reflecting differences in the productivity of both strains. Since females die in diet lacking tetracycline, double the amount of eggs from the TD1 strain were seeded onto 20 L trays containing this diet. We observed that similar numbers of insects were obtained in diet lacking tetracycline compared to those obtained from half the amount eggs reared in diet containing tetracycline (*P* = 0.825, One-Way ANOVA; Fig. 4c), suggesting that in the first case all of the insects obtained were male. To confirm this, we evaluated the number of males obtained under these mass rearing conditions. Indeed, we found that it was possible to obtain twice the number of TD1 males in diet without tetracycline when double the number of eggs were seeded in 20 L trays, with respect to males obtained in diet containing tetracycline (*P* < 0.001, One-Way ANOVA; Fig. 4d). The rates of emergence of insects from pupae remained high, between 81 and 97% and no females were obtained (additional file 4 a, b). Further, the number of TD1 males obtained in restrictive mass rearing conditions was similar to that obtained with the J06 strain, although with this wild type strain both sexes were produced (*P* = 0.787, One-Way ANOVA; Fig. 4d).

## Discussion

To develop strains where females die early in development using the tetracycline-off system, the promoters of embryonic cellularization genes have been used for expression of tTA, due to their high activity at the cellular blastoderm stage but low activity at later stages of development [18, 19, 24]. Consequently, for developing transgenic embryonic sexing strains of *L. cuprina*, we used the *Lsbnk* promoter as well as the promoter from *L. sericata spitting image* (*Lsspt*), a paralog of the zygotic cellularization gene *serendipity alpha* (*sry-α*) [20, 23]. Promoters from *Lucilia* cellularization genes were used because at the time this project began we had limited access to NWS material. However, with the recent assembly of the NWS genome [25], future tTA drivers could be made with promoters from NWS genes. Both *Lsbnk and Lsspt* promoters were active in early embryos but also active in adult *L. cuprina* females [20, 23]. Recently, tTA expression in ovaries was confirmed by crossing driver lines with a tetO-DsRed reporter strain [21]. A similar phenomenon was also reported in the early lethal strains of *Anastrepha ludens*, in which the promoter from *A. suspensa sry* (*Assry*) activated tTA expression in embryos as well as adult females, and non-vitellogenic ovaries were observed from females developed in diet lacking tetracycline [26]. In the DH sexing strains made in this study, females from two strains were fully sterile and one strain had significantly reduced fertility without an additional tetracycline supply in the adult water, suggesting that the *Lsbnk* promoter is active in NWS ovaries as was observed in *L. cuprina* [21]. Interestingly, females from the other DH strain and the TD1 strain were fertile and viable on diet that lacked tetracycline. This suggests that there is relatively low expression of tTA in the ovaries in these strains. Such differences in promoter activity in different strains is likely due to negative genomic position effects, which are associated with *piggyBac* mediated transformation [27, 28]. Therefore, a tetO-DsRed reporter strain could be considered for NWS, which will facilitate the future development of early lethal stains by identifying the driver lines that have little, if any, tTA expression in ovaries.

The All-in-one construct has some advantages such as ease of breeding to homozygosity as only a single transgene is being selected, and potential lower fitness costs, as each transgene insertion could incur a fitness cost. Further, for a fertile release program a single component system would appear to be advantageous as the transgenes for a two-component strain would separate after two generations of crossing with wild type. Another advantage of an All in one strain over the DH strains is that it requires less steps for “stabilization” by removing the *piggyBac* ends [29, 30], which could improve the stability during mass rearing and in the field for a fertile release. Interestingly, like the TD1 strain reported here, females for the two *L. cuprina* All-in-one early lethal strains reported previously did not need additional tetracycline supply in the adult drinking water and female offspring died at the embryo stage [20]. However, in these strains high mortality was also recorded in the male offspring. The males showed overexpression of the DsRed marker at the third instar larvae stage, which suggested that tTA bound to tetO was activating the linked tTA and DsRed genes [31]. Male lethality was thought to be due to the overexpression of tTA [20]. However, the NWS TD1 strain did not show increased DsRed fluorescence. Further, male production was similar on diet with or without tetracycline. Thus, it appears that the DsRed and tTA genes are not overexpressed in TD1#12 males. Our failure to maintain the other two independent TD1 lines could be due to a toxicity in both sexes that was not fully suppressed by tetracycline in the diet. Since a limited number of All-in-one strains were generated in *L. cuprina* and NWS, it is difficult to conclude if the performance of such a gene cassette is subjected to species or position effects. Nevertheless, the TD1 strain obtained here not only showed promising fitness characteristics that are critical for mass-rearing at larger scales (Fig. 3), but also resulted in significant male production and stable female lethality in the mass rearing experiments (Fig. 4). Considering that this strain produces lower numbers of insects than J06, it would be desirable to test the productivity of males when different numbers of eggs are seeded in large 20 L trays of larval diet without tetracycline to select the optimum egg number required to obtain the largest productivity of male insects maintaining the initial fitness characteristics observed previously in the laboratory. Consequently, this strain may be suitable for large-scale production and semi-field tests to further score its performance in a SIT program.

Currently, the presence of NWS in South America and some Caribbean islands continues to cause great economic damages to livestock production [11] and also poses a threat to areas where screwworm was present before the eradication program [12]. The COPEG program in Panama releases 15 million sterile flies per week in the Panama-Colombia border to effectively prevent the re-invasion of the NWS from South America [7]. In a recent SIT program to control the NWS outbreak in the Florida Keys, 154 million flies per week were released during a 6-month period to suppress emerging NWS populations and achieve their full eradication [12]. The cost of larval rearing of these screwworms in the mass-rearing facility is about US $1.0 per 1000 insects, which is already an improvement over the cost of ingredients used in the past [7, 10]. The transgenic strains developed in this study could significantly reduce the costs of a future eradication campaign. Modeling studies suggested that 2–70 males carrying a dominant female lethal gene achieves the same population suppression effect as 16–3000 SIT males [32]. Further, such a suppression effect can be enhanced if the males carry multiple female-lethal loci on separate chromosomes [33]. For the multiple loci strategy, the All-in-one gene cassette could be inserted into multiple sites using *piggyBac*-mediated transformation, or by precise gene editing mediated by CRISPR/Cas9 [34]. Since the late stage larvae consume most of the larval diet in a mass rearing plant [35], the early female lethal strains could provide significant savings in diet costs by removing the females from the rearing process at the first instar larvae stage. Thus, the early female lethal strains could increase the production capacity of the mass rearing plant and improve the efficiency of the control program. The next steps in the evaluation of these strains would be to study their mating behavior and other fitness characteristics relevant to field performance, and then develop one of the strains for a large-scale mass rearing program followed by a small-scale field release.

## Conclusions

We have generated and evaluated four two- component early female lethal strains and an All-in-one strain of NWS. All strains produced only males on a restrictive tetracycline feeding regimen. The females appear to die at embryo or first instar larvae stages. Following evaluation of fitness characteristics that are important for mass rearing, one two-component strain and the All-in-one strain appear to be particularly promising candidates to replace the current bisexual J06 strain that is used by the COPEG control program in Panama. The strains should significantly reduce costs of male production and increase population suppression efficiency through male-only releases.

## Methods

### Fly rearing and germline transformation

The J06 strain of *C. hominivorax* was collected in Jamaica in 2006 and is the wild type strain that is reared routinely at the COPEG biosecurity plant. Adult females are stimulated to lay eggs by presenting them with warm containers of raw ground meat mixed with an attractant made from spent larval media. Eggs are collected from adult females on the sixth day after emergence and are seeded in artificial larval diet (containing dry blood, dry egg, dry fishmeal protein, and cellulose fiber) and kept at 39 °C and 80% humidity for 3 days, adding more food daily, until they have reached the third instar larvae stage. On the fourth day of development, the larvae are placed in a room at 31 °C and 80% humidity for pupation into containers of sawdust. On the eighth day, the pupae are sieved out of the sawdust and placed into cages for emergence of adults in a colony room at 25.5 °C and 55% humidity. Insects were reared in a 12 h/12 h light/dark cycle. The J06 strain was used for *piggyBac* mediated germ-line transformation using a protocol similar to the one developed for *L. cuprina* [36]. Specifically, pre-blastoderm embryos were injected with a mix of pBac [DR2 or EF1 or All in one] plasmid (800 ng/μL), Lchsp83-pBac helper plasmid (400 ng/μL), and pBac RNA helper (400 ng/μL) [14, 31]. Two sources of helper were used as this appeared to increase the efficiency of *piggy Bac* mediated germline transformation (unpublished). A DNA template was prepared and in vitro synthesis of *piggyBac* RNA helper was performed as described previously [14, 31]. First instar larvae showing transient expression of the ZsGreen or DsRedex2 marker were selected and raised on diet supplemented with 200 μg/mL tetracycline. This is a relatively high dose of tetracycline, which may present a challenge in terms of cost of purchasing and diet waste disposal. G_0_ adults were crossed to wild type flies and their offspring were screened for expression of the fluorescent marker as first instar larvae. Homozygous *C. hominivorax* individuals were selected as crawl-off third instar larvae based on fluorescence intensity and bred to create a stable line.

### Female lethality tests and early lethality assessment

The double homozygous breeding was performed as previously described [20] and larvae were reared on tetracycline-free or 200 μg/mL tetracycline larval diet. To assess female lethality in a double heterozygous condition, 8 newly emerged males from a homozygous driver line and 8 newly emerged virgin females from a homozygous effector line were put in one bottle and kept on tetracycline-free adult diet for 8 days. Meat was provided for oviposition and embryos collected 24 h later. Larvae were reared on tetracycline-free larval diet, pupae collected and the number of adult males and females were counted. Female lethality in a double homozygous condition was addressed in the same way except newly emerged adults were fed water containing tetracycline (10 μg/mL) for the first 1 or 2 days and then switched to tetracycline free water. Embryos were collected as previously described and larvae were reared on free or 200 μg/mL tetracycline larval diet and the number of pupae, adult males and females was counted. For staged lethality tests, embryos were collected on ground beef and then transferred to a petri dish containing moist black filter paper and counted. Each petri dish held 1000 embryos. The number of hatched first instar larvae were counted and then transferred to larval diet. The number of first, second and third instar larvae, pupae, adult males and females were monitored. All lethality tests were done in triplicate.

### Fitness essays

Fitness tests were performed for all the transgenic lines and for the J06 parental wild type strain, according to protocols used regularly in the COPEG biosecurity facility for quality control [14]. All tests were replicated at least three times unless otherwise indicated.

### Survival from eggs to adults

For each transgenic line and for the J06 strain, 75 mg of eggs were seeded in larval diet and raised until the pupal stage. The total volume of pupae was measured using a graduated cylinder and the total number of pupae in 25 mL was counted. From this, the total number of pupae and the average pupal weight was calculated. Regular testing has established that 75 mg of eggs is equal to 1875 eggs, and therefore the percentage of eggs that develop into pupae can be calculated.

### Fertility

To measure the fertility of each line, eggs were collected and the egg mass dissociated into individual eggs by incubating them in a 4% w/v sodium hydroxide solution for 2 min with constant stirring and then rinsing them with abundant distilled water. For each line, 300 individual eggs were placed in a petri dish containing a damp paper towel and black filter paper on top. The petri dishes were incubated at 37 °C overnight and, the following morning, the number of hatched larvae were counted and the percentage egg hatch was calculated.

### Fecundity

For each transgenic line and the J06 strain, a cage was set up with 50 males and 50 females with food and water containing tetracycline. On the sixth day after emergence, the females were induced to lay eggs and the total weight of the egg masses was measured. The average total number of eggs laid per female was calculated by dividing the total egg weight in mg by the number of females in the cage and then multiplying by 25, as 100 mg of eggs equals approximately 2500 eggs.

### Adult emergence and sex ratio

For each transgenic line and a J06 control, 100 pupae were placed in a closed container and adults were allowed to emerge for 3 days after the emergence of the first insect. Males and females were counted and percentage of emergence and sex ratio calculated.

### Fitness under mass rearing conditions

Approximately 36,000 eggs were seeded in 20 L of larval diet in a large polystyrene tray and incubated at 39 °C and 80% humidity for 3 days, adding more food daily, until they reached the third instar larvae stage. On the fourth day of development, the larvae were placed in a room at 31 °C and 80% humidity for pupation into containers of sawdust. On the eighth day, the pupae were sieved out of the sawdust and the total volume of pupae was measured. Fitness tests corresponding to hatching, average pupa weight, emergence and sex ratio were performed as previously described [14].

### Statistics

All statistics were carried out using SigmaPlot v12.5 (Systat Software). The differences in the fly number (numbers from certain test were log transformed to make the variance independent of the mean), average pupae weight, hatching rate and pupae development rate from multiple strains were tested by one-way analysis of variance (ANOVA) and means were separated using either Holm-Sidak method or Duncan’s multiple range test. The fly number from two sources was analyzed using two-sample t-tests as were the mean values from either two different strains under the same tetracycline condition or the same strain under different tetracycline conditions.

## Supplementary Information

**Additional file 1. **Assessment of female viability with and without tetracycline in the diet. **a** Raw data from heterozygous female lethality tests performed for all the early female lethal strains studied. **b** Raw data from homozygous female lethality tests performed for all of the transgenic strains that survived after rearing for several generations in the laboratory.

**Additional file 2.** Tetracycline dosage studies for two double homozygous early lethal strains. Specific fitness characteristics for insects reared in diet containing 100, 150, 200 and 300 μg/mL tetracycline to assess the concentration that yields the highest fitness parameters for mass rearing. We studied the average number of eggs laid, the percentage of adult development from eggs (Biological yield), the average pupae weight and the sex ratio for the (A) DR2–16-EF1-6B and (B) DR2–17-EF1–16 strains.

**Additional file 3.** Tetracycline dosage in drinking water affects egg laying of females. To find the best conditions for obtaining good amounts of eggs from the adult double homozygous females, we studied the weight of the egg mass laid in cages of adult insects reared with no tetracycline in the water and with 10 μg/mL tetracycline in the water given for one day, two days and permanently in four transgenic strains.

**Additional file 4.** Fitness of TD1 strain under mass rearing conditions. **a** Percentage of insects that emerge from pupae. **b** Sex ratio. Number of males and females emerged from 100 pupae.

## Data Availability

The datasets supporting the conclusions of this article are included within the article and its additional files.

## Abbreviations

COPEG: Comisión Panamá - Estados Unidos para la Erradicación y Prevención del Gusano Barrenador del Ganado
SIT: Sterile insect technique
