# Supplementary material for: An early female lethal system of the New World screwworm, Cochliomyia hominivorax, for biotechnology-enhanced SIT
Source: BMC Genet. 2020 Dec 18;21(Suppl 2):143. doi: 10.1186/s12863-020-00948-x (PMC7747452; doi:10.1186/s12863-020-00948-x)
Supplement: Supplementary file 1 — Additional file 1. Assessment of female viability with and without tetracycline in the diet. a Raw data from heterozygous female lethality tests performed for all the early female lethal strains studied. b Raw data from homozygous female lethality tests performed for all of the transgenic strains that survived after rearing for several generations in the laboratory. [file 12863_2020_948_MOESM1_ESM.pdf]

Additional file 1.
Heterozygous female lethality in two-component Driver-Effector combinations.

HETEROZYGOUS TRANSGENIC LINES									
**Driver**	**Effector**	**TET**	**Bottle #**	**# pupae**	**# male**	**# female**	**% female**	**Average**	**StDev**
**DR2-4**	**EF1-16**	NO	1	244	206	26	11.21%	6.57%	4.76%
			2	191	173	6	3.35%		
			3	227	201	12	5.63%		
			4	173	124	18	12.68%		
			5	54	49	0	0.00%		
		YES	1	26	11	13	54.17%	47.66%	6.37%
			2	249	129	82	38.86%		
			3	378	165	188	53.26%		
			4	342	172	137	44.34%		
**DR2-8**	**EF1-16**	NO	1	125	91	0	0.00%	0.00%	0.00%
			2	37	34	0	0.00%		
			3	44	44	0	0.00%		
			4	84	69	0	0.00%		
			5	122	120	0	0.00%		
			6						
		YES	1	605	294	298	50.34%	49.99%	0.34%
			2	154	72	71	49.65%		
			3						
			4						
			5						
**DR2-11A**	**EF1-16**	NO	1	290	241	1	0.41%	0.35%	0.41%
			2	364	349	0	0.00%		
			3	238	184	0	0.00%		
			4	291	197	2	1.01%		
		YES	1	502	180	180	50.00%	50.12%	0.12%
			2	693	309	312	50.24%		
			3						
			4						
**DR2-11B**	**EF1-16**	NO	1	28	28	0	0.00%	0.00%	0.00%
			2	41	41	0	0.00%		
			3	52	48	0	0.00%		
			4						
		YES	1	156	78	72	48.00%	50.03%	1.83%
			2	320	147	145	49.66%		
			3	361	166	183	52.44%		
			4						
**DR2-17**	**EF1-16**	NO	1	166	114	0	0.00%	0.00%	0.00%
			2	160	156	0	0.00%		
			3	180	151	0	0.00%		
			4	179	124	0	0.00%		
			5	190	175	0	0.00%		
			6						
		YES	1	425	167	203	54.86%	49.86%	3.55%
			2	106	46	42	47.73%		
			3	107	44	39	46.99%		
			4						
			5						
			6						

**DR2-21**	**EF1-16**	NO	1	88	80	0	0.00%	0.85%	1.21%
			2	80	76	2	2.56%		
			3	25	25	0	0.00%		
			4						
		YES	1	114	57	43	43.00%	48.97%	5.97%
			2	93	41	50	54.95%		
			3						
			4						
**DR2-17**	**EF1-19**	NO	1	186	147	31	17.42%	13.65%	6.04%
			2	200	124	28	18.42%		
			3	41	37	2	5.13%		
			4						
		YES	1	855	592	218	26.91%	36.50%	7.62%
			2	392	192	113	37.05%		
			3	108	55	46	45.54%		
			4						
**DR2-17**	**EF1-6B**	NO	1	136	122	16	11.59%	8.36%	5.96%
			2	181	122	19	13.48%		
			3	60	56	0	0.00%		
			4						
		YES	1	232	92	109	54.23%	52.69%	3.23%
			2	119	43	40	48.19%		
			3	190	55	69	55.65%		
			4						
**DR2-21**	**EF1-6B**	NO	1	48	35	0	0.00%	0.00%	0.00%
			2	44	38	0	0.00%		
			3	48	36	0	0.00%		
			4						
			5						
		YES	1	178	65	86	56.95%	53.89%	3.07%
			2	150	120	124	50.82%		
			3						
			4						
			5						
**TD1**		NO	1	339	296	0	0.00%	0.00%	0.00%
			2	294	261	0	0.00%		
			3	330	298	0	0.00%		
		YES	1	483	219	201	47.86%	48.30%	0.46%
			2	522	215	206	48.93%		
			3	560	219	203	48.10%		
**J06**		NO	1	715	358	335	48.34%	48.59%	0.38%
			2	789	348	336	49.12%		
			3	770	380	355	48.30%		
		YES	1	667	377	266	41.37%	46.29%	11.24%
			2	289	157	87	35.66%		
			3	951	329	533	61.83%		

Homozygous female lethality in two-component Driver-Effector combination.

HOMOZYGOUS TRANSGENIC LINES									
Driver	Effector	TET	Bottle #	# pupae	# male	# female	% female	Average	StDev
DR2-8	EF1-16	NO	1	122	81	7	7.95454545	9.94506548	3.76340984
			2	25	18	3	14.2857143		
			3	99	73	6	7.59493671		
		YES	1	151	55	75	57.6923077	51.7325017	5.77866597
			2	102	56	48	46.1538462		
			3	39	18	19	51.3513514		
DR2-11A	EF1-16	NO	1	236	105	0	0	0	
			2	240	114	0	0		
			3	167	129	0	0		
			4	144	117	0	0		
		YES	1	273	97	109	52.9126214	51.434796	2.25715118
			2	278	139	131	48.5185185		
			3	420	180	186	50.8196721		
			4	275	100	115	53.4883721		
DR2-17	EF1-16	NO	1	141	134	0	0	0	
			2	255	237	0	0		
			3	111	91	0	0		
		YES	1	206	91	72	44.1717791	46.2956679	1.87612005
			2	106	46	42	47.7272727		
			3	107	44	39	46.9879518		
DR2-21	EF1-16	NO	1	244	206	0	0	0.37664783	0.65237319
			2	105	81	0	0		
			3	237	175	2	1.1299435		
			4						
		YES	1	352	122	127	51.0040161	49.9637057	2.63279998
			2	388	175	155	46.969697		
			3	472	163	176	51.9174041		
			4						
DR2-17	EF1-6B	NO	1	225	189	0	0	0	
			2	315	263	0	0		
			3	247	181	0	0		
			4	176	154	0	0		
		YES	1	369	103	135	56.7226891	53.2325857	3.85230289
			2	445	119	139	53.875969		
			3	283	113	109	49.0990991		
			4						
TD1		NO	1	266	45	0	0	0	
			2	155	119	0	0		
			3	158	101	0	0		
		YES	1	247	86	68	44.1558442	46.3361533	5.36453242
			2	387	182	134	42.4050633		
			3	194	68	75	52.4475524		
J06		NO	1	358	335	48.3	12.6010957	12.435132	0.42295495
			2	348	336	49.1	12.7499351		
			3	380	355	48.2	11.9543651		
		YES	1	377	266	35.8	11.8621604	17.0965759	10.3673234
			2	157	87	35.6	29.0375204		
			3	329	533	61.8	10.3900471		
