# Supplementary material for: An early female lethal system of the New World screwworm, Cochliomyia hominivorax, for biotechnology-enhanced SIT
Source: BMC Genet. 2020 Dec 18;21(Suppl 2):143. doi: 10.1186/s12863-020-00948-x (PMC7747452; doi:10.1186/s12863-020-00948-x)
Supplement: Supplementary file 2 — Additional file 2. Tetracycline dosage studies for two double homozygous early lethal strains. Specific fitness characteristics for insects reared in diet containing 100, 150, 200 and 300 μg/mL tetracycline to assess the concentration that yields the highest fitness parameters for mass rearing. We studied the average number of eggs laid, the percentage of adult development from eggs (Biological yield), the average pupae weight and the sex ratio for the (A) DR2–16-EF1-6B and (B) DR2–17-EF1–16 strains. [file 12863_2020_948_MOESM2_ESM.pptx]

## Slide 1
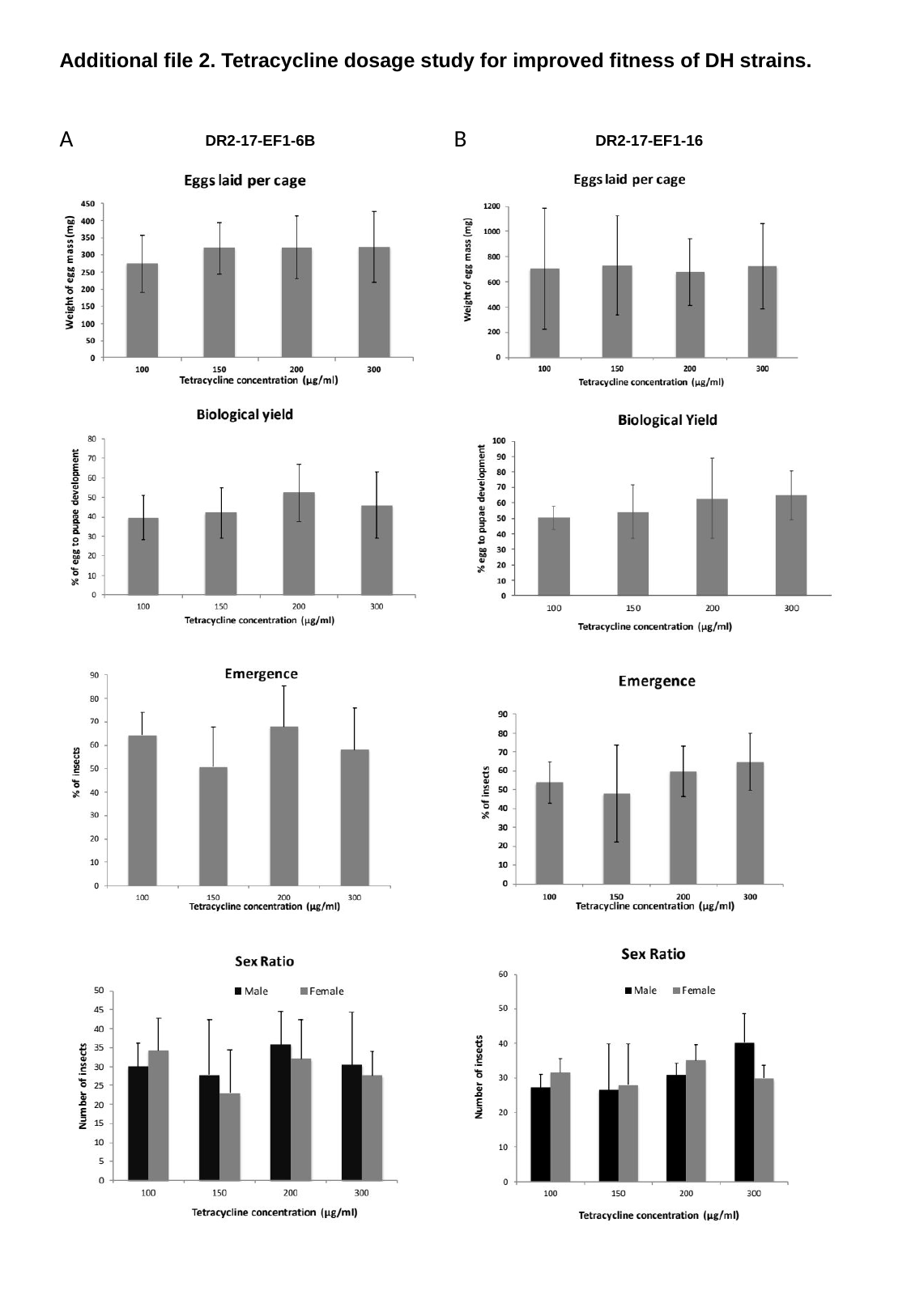

Additional file 2. Tetracycline dosage study for improved fitness of DH strains.
B
A
 DR2-17-EF1-6B DR2-17-EF1-16
