# Supplementary figures and images for: An early female lethal system of the New World screwworm, Cochliomyia hominivorax, for biotechnology-enhanced SIT
Source: BMC Genet. 2020 Dec 18;21(Suppl 2):143. doi: 10.1186/s12863-020-00948-x (PMC7747452; doi:10.1186/s12863-020-00948-x)

## Slide 1
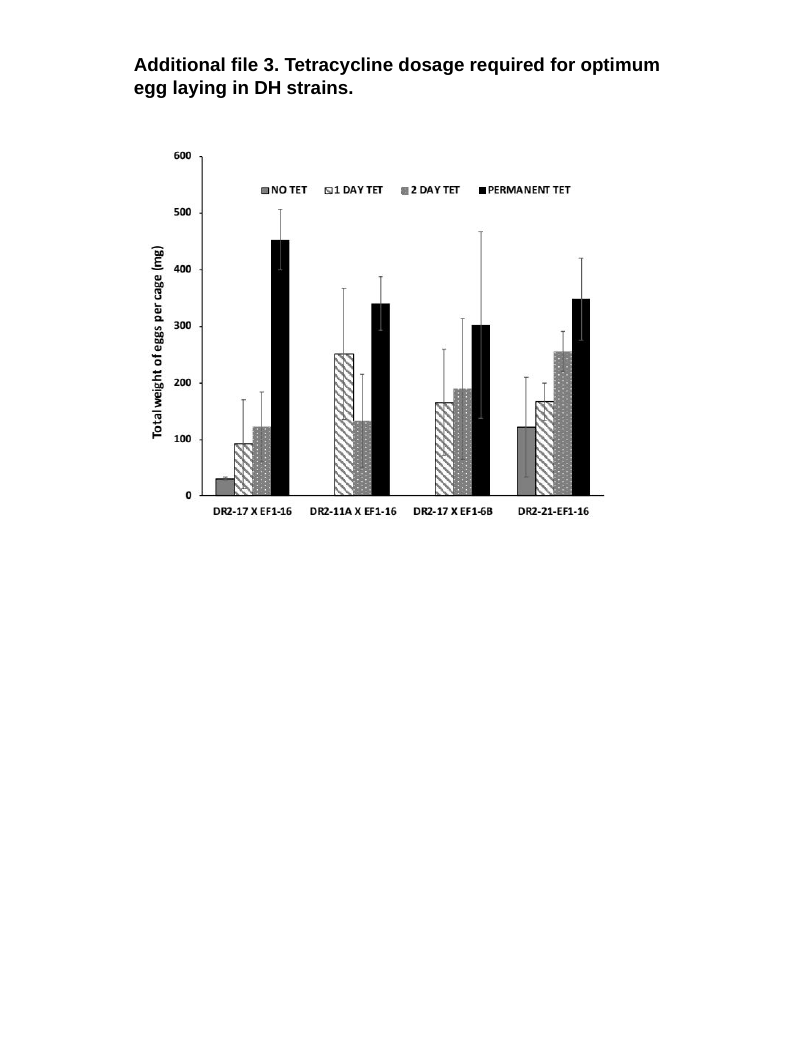

Additional file 3. Tetracycline dosage required for optimum
egg laying in DH strains.

Supplement: Supplementary file 3 — Additional file 3. Tetracycline dosage in drinking water affects egg laying of females. To find the best conditions for obtaining good amounts of eggs from the adult double homozygous females, we studied the weight of the egg mass laid in cages of adult insects reared with no tetracycline in the water and with 10 μg/mL tetracycline in the water given for one day, two days and permanently in four transgenic strains. [file 12863_2020_948_MOESM3_ESM.pptx]

## Slide 1
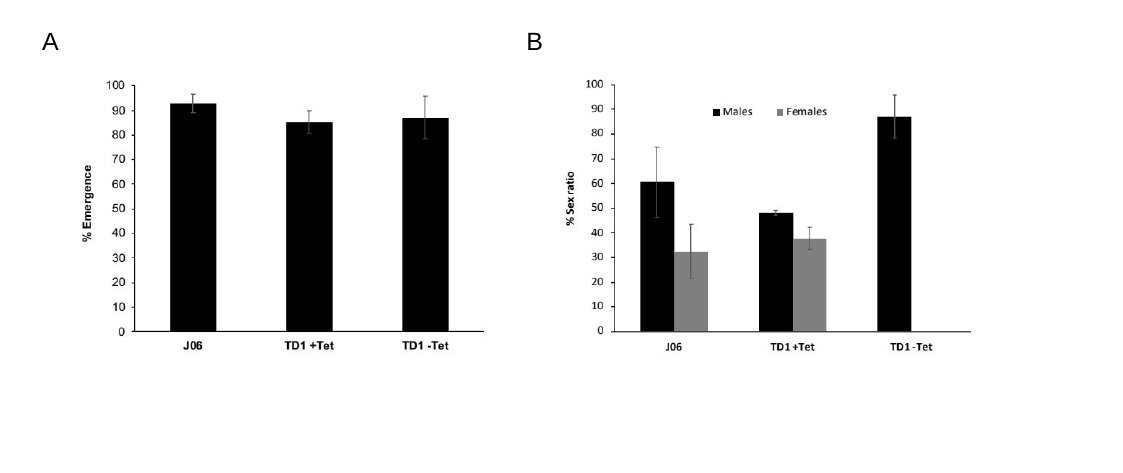

A
B

Supplement: Supplementary file 4 — Additional file 4. Fitness of TD1 strain under mass rearing conditions. a Percentage of insects that emerge from pupae. b Sex ratio. Number of males and females emerged from 100 pupae. [file 12863_2020_948_MOESM4_ESM.pptx]
